# Survey on urban transport in the aftermath of the COVID-19 outbreak: Data from 20 cities across Europe

**DOI:** 10.1016/j.dib.2023.108910

**Published:** 2023-01-16

**Authors:** Elena Navajas-Cawood, Davide Fiorello, Mari Angeles Martinez, Juan Vicente Castellanos Quintana, Gabriella Scarcella, Panayotis Christidis

**Affiliations:** a European Commission^1^, Joint Research Centre, Seville, Spain; b TRT Transporti e Territorio, Milan, Italy; c IPSOS^2^, Barcelona, Spain; d IPSOS, Madrid, Spain; e IPSOS^3^, Milan, Italy

**Keywords:** Pandemic, Mobility, Impact analysis, User choices, Car use, Public transport

## Abstract

This dataset contains the full results of a survey on mobility patterns after the Covid-19 pandemic. The survey was conducted in the second trimester of 2021 and collected information from 10000 respondents across 20 urban areas. The questions covered demographic and socio-economic characteristics, employment and job related situation, the use of technological alternatives in daily activities, mobility patterns (trip frequency, purpose, destination, mode, level of comfort), and perceptions as regards the usability of each transport option. Particular emphasis was given to the comparison of current activity to that before the pandemic. The survey combined an online (Computer-Assisted Web Interviews, CAWI) approach with telephone (Computer-Assisted Telephone Interviews, CATI) interviews. The sample in each city was representative of the local demographic and socio-economic profile according to age, sex, employment situation, education and urbanization.

**Specifications Table**

**Table:** 

Subject	Transportation Management
Specific subject area	Impacts of COVID-19 pandemic on urban transport and mobility choices
Type of data	Table • full dataset of responses to all questions, as a table with 10000 rows and 284 columns (“raw_data.csv”) • table of correspondence between questions, variables in dataset, and answer options (data dictionary as 2 Excel tables, “data_specifications.xlsx”) • English version of the full questionnaire used in the survey, as a text document (“Full questionnaire.docx”)
How the data were acquired	Survey in 20 cities (Functional Urban Areas) across Europe. Combined approach of online (Computer-Assisted Web Interviews, CAWI) and telephone (Computer-Assisted Telephone Interviews, CATI) interviews. 80% of respondents through CAWI, 20% of respondents through CATI.
Data format	Raw
Description of data collection	For the CAWI survey, randomly selected participants received an invitation email with the survey description, instructions and privacy policy. Sampling was carried out via a proprietary sampling application allowing to construct complex samples based on the target and screening requirements. The CATI sample was randomly extracted from databases of phone landlines and was integrated into the CAWI sample to ensure that all relevant segments are represented. Quotas were applied on the target number of respondents per socio-economic group. For each city, the target share of respondents matched its population profile according to five variables: age, sex, employment situation, education and urbanization.
Data source location	500 respondents each in the following 20 cities: Charleroi (Belgium); Prague, Brno (Czechia); Paris, Lille (France); Berlin, Dresden (Germany); Dublin (Ireland); Milan, Catania (Italy); Krakow, Poznan (Poland); Lisbon, Porto (Portugal); Bacau, Cluj-Napoca (Romania); Madrid, Málaga (Spain); Stockholm, Malmö (Sweden)
Data accessibility	https://dataverse.harvard.edu/dataset.xhtml?persistentId=doi:10.7910/DVN/VVXHQA 10.7910/DVN/VVXHQA
Related research article	P. Christidis, E.N. Cawood, D. Fiorello, Challenges for urban transport policy after the Covid-19 pandemic: Main findings from a survey in 20 European cities, Transport Policy. 129 (2022) 105–116. 10.1016/j.tranpol.2022.10.007. https://www.sciencedirect.com/science/article/pii/S0967070X2200292X?via%3Dihub

**Value of the Data**

• Useful information for research on individual choices as regards urban mobility after the pandemic.
• Data allow association of mobility preferences with socio-economic and demographic characteristics.
• Representative sample allows comparisons between cities and can be combined with other sources for further international evaluations.
• Sampling efficiency at Functional Urban Area (FUA) level allows combination with spatial data and econometric analyses.
• Results can be used for the exploration of changes in teleworking, online shopping and other alternatives to physical trips
• The indication of whether a respondent was included in the CAWI and CATI sample can be useful for the analysis of mode effects (web versus phone interviews)
• The dataset can serve for educational purposes in survey analysis and classification techniques

## 1 Objectives

The main objective of the survey was to identify the changes in activity, lifestyles and preferences triggered by the Covid-19 pandemic which may lead to a prolonged effect on the urban transport system, especially as regards demand and modal split. The survey identified the changes introduced by the coronavirus pandemic in transport habits, as well as citizens’ potential new expectations arising from this situation regarding public transport policy and urban planning. Since the focus of the survey was urban transport, the fieldwork was conducted in 20 urban areas within the EU, ensuring representative samples that cover a wide variety of city profiles. The published article linked to this dataset [1] describes the main findings and explore the kay factors that explain changes in mobility. The full dataset of the survey is made available for replication purposes and as a source of data for further research.

## 2 Data Description

The survey was carried out in 20 cities across 11 Member States of the European Union during the 2^nd^ trimester of 2021 and addressed the impacts of the pandemic on personal mobility choices. The goal was to identify the changes in activity, lifestyles and preferences that may lead to a prolonged effect on the urban transport system, especially as regards demand and modal split. The structure of the questionnaire was based on previous research discussing the main challenges for transport in the aftermath of the Covid-19 pandemic [2], [3], [4]. The survey explored the changes in mobility, as well as citizens’ potential new expectations arising from this situation regarding public transport policy and urban planning.

For each city, 500 respondents completed a detailed questionnaire:

• Demographic information: age, gender, level of education, income, number of household members
• Employment information: employment status, change of employment status due to pandemic, frequency of teleworking, reasons for not being able to telework
• Mobility patterns before the pandemic:
  ○ Frequency of use of transport avoidance options: work from home, video calls to family and friends, phone/video-conferencing for work, use of phone and video calls for health and medical services, purchase of goods online, use of home delivery services for groceries
  ○ Frequency of use of transport modes: walking, private bike/ e-bike, shared bike/ e-bike, private scooter/ e-scooter, shared scooter services, private motorbike/ moped, shared motorbike/ moped, private car as driver, private car as passenger, shared car as driver, shared car as passenger, taxi, ride-hailing services, urban public transport (bus, tram, metro, rail, etc.)
  ○ Frequency of use of each mode for each trip purpose: commuting, business, education, visiting relatives/ friends, accompanying children to/from school, accompanying family and friends, purchasing groceries, other shopping, leisure
• Specific changes of mobility patterns due to the pandemic: use of transport avoidance options, level of comfort with using transport avoidance options, type of change in mobility patterns (number of trips, transport mode, trip schedule, destination), trip purposes affected, frequency of use of each mode, reasons for change in most frequent mode used
• User expectations as regards future mobility patterns: change in frequency of use of transport avoidance options, change in frequency of each transport mode
• Change in vehicle ownership due to the pandemic: purchase of cars (new, second-hand), bicycles, electric bicycles, motorbikes/ mopeds, scooters/ e-scooters
• Accessibility of public transport: distance, availability, frequency, changes due to the pandemic

The dataset is used in [1] and can be freely used for replication or further research. The supplementary material accompanying this article include:

• the English version of the full questionnaire used in the survey, as a text document (“Full questionnaire.docx”)
• the full dataset of responses to all questions, as a table with 10000 rows and 284 columns (“raw_data.csv”)
• The table of correspondence between questions, variables in dataset, and answer options (data dictionary as 2 Excel tables, “data_specifications.xlsx”)

## 3 Experimental Design, Materials and Methods

The survey was be carried out in 20 cities across 11 European countries and addressed individuals aged 16 to 74 years old residing in the respective Functional Urban Area –FUA [5]. The development of the questionnaire followed the approach by [6] and involved several phases. The first phase consisted of a draft version in English, which was discussed extensively with transport experts and policy stakeholders. The draft version of the questionnaire was translated into the local languages in the cities covered by the survey, and was evaluated in a testing phase (Q-test) in order to test whether the way questions were asked was clear and understood as expected by the different profiles of potential interviewees. More precisely, the role of this phase is checking that questions are understandable in terms of how they are conceived, i.e. that they are interpreted as intended, that there are not ambiguous cases among the response options or situations that do not fall in each of the alternatives. After the Q-test, the questionnaire was updated in all language versions and translated back to English, in order to ensure that the formulation used in each language has the same meaning.

A specific tool (Ipsos MR Translator) was used for the implementation of the questionnaire. The tool guarantees a high quality of the results, optimising and speeding up the process. In particular, it helps to avoid mismatches between the master and the subsequent languages using a common database structure. The script of the questionnaire (i.e. screening questions, question positions, error messages, etc.) followed the same structure for all languages in order to avoid errors and inconsistencies, and to produce a final unique dataset for both CAWI and CATI interviews.

Using the scripted online version, a pilot test with 30 residents in each city was conducted, in order to validate aspects such as the duration of the interview, the internal consistency of the responses and the format of the output.

The actual fieldwork started with the CAWI survey. Randomly selected participants received an invitation email with the following information: short survey description, duration, a unique URL that provides access to the questionnaire, the physical address of the survey coordinator, support email address/link, link to privacy policy and instructions for opting-out.

Using the panels, sampling began by developing targets to match the sampling quotas.

Sampling was carried out via a proprietary sampling application allowing to construct complex samples based on the target and screening requirements. For this study the sampling groups were based on age, gender, employment condition, education level and geographical location. This last element concerns the location of respondents either in the major cities of the FUA or in its commuting area: the sample ensured that at least a certain share of respondents live in other municipalities than the main city of the FUA.

The software uses an interactive selection algorithm that balances one variable at a time in order of priority, as follows:

• The first step is to extract all active and available panellists that meet the screening criteria (ex. demographic, geographic).
• The sample pool is randomly sorted.
• The algorithm then examines the first (primary) variable and selects the number of survey respondents who satisfy each target. (Sometimes, there may not be enough available sample to fill all cells and since some variables are more important than others, lower priority variables may not balance precisely).
• Finally, the sample may be distributed and balanced among more than one cell so that different treatments or surveys may be fielded in equal balanced groups or cells.

The CATI sample was randomly extracted from databases of phone landlines and was integrated into the CAWI sample to ensure that all relevant segments are represented. During the fieldwork, specific quotas with reference to four segmentation elements were defined:

• Age and gender.
• Level of education.
• Employment status.
• Type of municipality (core or not).

For establishing the quota distribution, we consulted official data provided by Eurostat [7] and OECD [8].

We followed a statistical procedure to adjust the representativeness of each individual of the sample in order to use the sample data for estimating population values. Namely, each individual of the sample receives a weight consisting of the ratio between the share of the group the individual belongs in the population and the share of the same group in the sample. For example: if *U* is the share of females in reference population, *S* is the share of females in the sample the weight W associated to females in the sample is computed with the simple formula W = U / S. When the segmentation concerns several variables, like in this survey, the weight associated to each individual depends on the structure of the population and of the sample according to all the segmentation variables.

One aspect to be considered is that the distribution of the population for the combination of all variables together is unknown. To address it, weights are computed through a procedure controlling for the distribution according to each of the segmentation variables. Each record of the sample is identified by four segmentation variables:

• Age/gender (g)
• Employment status (m)
• Education level (d)
• Living area (r)

For each country and for each of these variables, the distribution of the population and the distribution of the sample is known. The following indicators are therefore available:

$PS_{g}$: share of age/gender group *g* in the population
$PS_{m}$: share of employment status *m* in the population
$PS_{d}$: share of education level *d* in the population
$PS_{r}$: share of living area *r* in the population
$SS_{g}$: share of age/gender group *g* in the sample
$SS_{m}$: share of employment status *m* in the sample
$SS_{d}$: share of education level *d* in the sample
$SS_{r}$: share of living aera *r* in the sample

Weights are based on the ratio between population and sample composition for the different stratification variables:

(1) $$W_{gmdr}=\;\frac{PS_{g}}{SS_{g}}\times \;\frac{PS_{m}}{SS_{m}}\;\times \;\frac{PS_{d}}{SS_{d}}\;\times \frac{PS_{r}}{SS_{r}}\;$$

So, all records of the sample (for one given country) belonging to age/gender group *g*, employment status *m*, education level *d* and living area *r* get the weight $W_{gmdr}$

If the distribution of population for all segmentation variables crossed together were known, weights could be computed based on population and sample shares, i.e.:

(2) $${\hat{W}}_{gmdr}=\;\frac{PS_{gmdr}}{SS_{gmdr}}\;$$

Where:

$PS_{gmdr}$: share of individuals of age/gender group *g*and employment status *m*and education level *d*and living area *r* in the population.
$SS_{g}$: share of individuals of age/gender group *g*and employment status *m*and education level *d*and living area *r* in the sample.

Weights computed in Eq. (1) coincide with the theoretical ratios (Eq. (2)) if the distribution of population according to one variable is the same in each group based on other variables, e.g.:

the share of individuals of age/gender group *g*and employment status *m*and education level *d* is the same in each region *d*,
the share of individuals of employment status *m*and education level *d*and living in region *d* is the same for each age/gender group *g*,

etc., for all combinations. If some groups are distributed in a significantly different manner according to another segmentation variable (e.g., if the share of graduate and non-graduate of employed individuals of a certain age/gender group *g* is different among regions *d*), aggregated weights computed with equation Eq. (1) will tend to diverge from the theoretical ratios Eq. (2). Weights are meaningful for each FUA independently, but do not serve for comparisons or aggregation across cities.

The resulting weighting efficiency of 80% on average was satisfactory, an indicator that suggests that the sample distribution was similar to the population distribution and overall, the weight of each respondent is reasonable. Nevertheless, in a few FUAs there were some individuals with a high or a low weight due to the distribution of the sample, especially for Romanian and Portugal FUAs where, it was difficult to reach some population segments. Analysing the weight, is important to highlight that 84.8% of the sample has a weight within the range between 0.5 and 1.99. It is noteworthy that in 7 FUAs all the individuals have a weight within this range: Madrid, Paris, Stockholm, Malmö, Dublin, Praha and Charleroi. In Dresden almost all cases (496) have a weight in the range of 0.5 to 1.99. These FUAs are the ones with higher weighting efficiency (between 93% and 98%). On the other hand, higher weight was assigned to men from the higher age segment with low level of education. However, less than 3% of the sample has a weight larger than 2; 2.5% of the sample has a weight between 2 and 4, and only the 0.3% of cases have a weight higher than 4. As Regards low weights, 10.6% of sample has a weight between 0.49 and 0.25 and 1.8% has a weight lower than 0.25. The highest weights (>4) are basically concentrated in the FUAS of Cluj-Napoca and Bacau (16 cases out of 17). And consequently these FUAs are the ones with lower weighting efficiency.

Table 1 summarizes the differences between the shares in the sample and the shares in the population. The most frequent category is used for each of the five weighting variables. Table 1 also includes the summary statistics on weighting efficiency and respondent weights. FUAs with a higher efficiency are those with weights close to 1.

**Table 1:** Differences between sample and population distribution (in percentage points) and summary statistics for each FUA covered by the survey.

	Age	Gender	Employment	Educational level	Living region			
	*35-54 years*	*Female*	*Employed*	*ISCED 0-4*	*Core*	Weighting efficiency	max respondent weight	min respondent weight
Bacau	0.5	6.4	1.3	-29.6	7.7	51.7%	5.2	0.16
Berlin	3.6	5.5	0.2	-25.3	12	79.7%	2.87	0.25
Brno	3.7	4.7	-2	-14	12.5	87.5%	2.23	0.46
Catania	5.1	7.9	8.5	-10.8	1.8	85.9%	2.64	0.38
Charleroi	2.6	5.7	6.2	-5.6	-2.5	93.9%	1.72	0.59
Cluj-Napoca	4.1	-0.4	2.9	-38.9	4	46.0%	6.72	0.18
Dresden	2.8	5.4	0.7	-21.6	1.7	92.5%	1.72	0.48
Dublin	1.5	1.5	3.5	-0.7	25.9	95.3%	1.61	0.72
Krakow	3.6	8.3	8.1	-20.2	31.6	82.6%	2.28	0.43
Lille	5.6	9.9	7.2	-18.7	14	82.8%	2.16	0.44
Lisbon	5.9	0.9	5	-25.1	18	70.2%	3.2	0.36
Madrid	2	0.2	-3.8	-7.3	0.8	97.7%	1.2	0.72
Malaga	5.4	6.4	6.5	-15.4	-1.4	84.0%	2.63	0.51
Malmo	0.1	3.1	-2.7	-12.2	2	94.2%	1.43	0.63
Milano	0.1	6.6	2.9	-15.2	31.5	87.9%	1.55	0.34
Paris	-1.5	-0.5	0.2	-12.5	0.5	93.8%	1.39	0.59
Porto	3.9	-3	4.5	-26.5	1.2	72.0%	2.76	0.33
Poznan	3.9	11.8	20.5	-20.9	33.1	71.7%	4.78	0.31
Prague	-4.5	2.6	-2.5	-21.8	15.5	93.2%	1.49	0.51
Stockholm	-0.1	2.4	-4.2	-6.4	0.7	97.7%	1.23	0.7

## Ethics Statements

All respondents confirmed their consent in participating in the survey. Data have been anonymized to protect personal information. The survey process and the resulting database are compliant with the EU's General Data Protection Regulation [9]. The internal Ethics Appraisal report for the European Commission, Joint Research Centre Project Number: 30432 was registered on 26/4/2022 (ARES (2021) 2772722).

## CRediT Author Statement

**Elena Navajas-Cawood:** Conceptualization, Methodology, Project Management, Supervision, Writing – review & editing; **Davide Fiorello:** Methodology, Project Management, Supervision, Data Analysis, Writing- Reviewing and Editing; **Mari Angeles Martinez:** Methodology, Data Collection, Data Analysis, Writing – review & editing; **Juan Vicente Castellanos Quintana:** Methodology, Data Analysis, Quality Control, Writing – review & editing; **Gabriella Scarcella:** Methodology, Writing – review & editing; **Panayotis Christidis:** Supervision, Conceptualization, Data Analysis, Methodology, Writing – review & editing.

## Declaration of Competing Interest

The authors declare that they have no known competing financial interests or personal relationships that could have appeared to influence the work reported in this paper.

## Data Availability

Survey on urban transport in the aftermath of the COVID-19 outbreak (Original data) (Dataverse).
